# "The Hospital" Nursing Mirror

**Published:** 1896-04-04

**Authors:** 


### "The Hospital" Nursing Mirror.

Being the Extra Nursing Supplement of "The Hospital" Newspaper.

[Contributions for this Supplement should be addressed to the Editor, The Hospital, 428, Strand, London, W.C., and should have the word "Nursing" plainly written in left-hand top corner of the envelope.]

### News from the Nursing World.

### THE DUKE AND DUCHESS OF YORK IN THE NORTH.

During their stay at Knowsley the Duke and Duchess of York took the opportunity to visit the Cottage Homes at Fazakerley, an experiment started by the West Derby Union for the up-bringing of pauper children amid home surroundings. The children of this poor-law village are some 500 in number, and all assembled, drawn up in rank, outside the gates to welcome the Royal visitors, and sang the National Anthem in greeting. Several members of the West Derby Board of Guardians were present, including Alderman Houlding, who is himself largely responsible for the scheme. When the Duke and Duchess drove off, after accepting from the Alderman a copy of the last report, the children gave three hearty cheers for "Our future King and Queen."

### NURSING POLITICS.

The cleavage between the disputants in the British Nurses' section of nursing politics may be regarded as complete. The discussion on the Barlow case in January and Sir Dyce Duckworth's article in The Hospital of February 8th plainly revealed the true position of affairs. These were followed by the announcement that H.R.H. the Princess Christian and Lady Jeune had retired from all connection with the Home of Rest for Nurses. Now the air is filled with rumours concerning the Registered Nurses' Society. We are informed that most, if not all, of the committee have resigned, and that a large number of the nurses have seceded. A new organisation, the Chartered Nurses' Society, of which full particulars are given elsewhere, has been started, with a committee on which appear the names of at least half of the members of the original committee of the Registered Nurses' Society. It is certainly instructive to remember that the few individuals who have brought about these serious differences and splits in the ranks of the British nurses' section of nursing are the same persons who seceded from the Hospitals Association years ago, on the ground that they could not work harmoniously in an association which consisted of laymen, doctors, and nurses. The only perfect organisation where harmony could be secured in conjunction with a due regard for the interests of nurses was one where doctors and nurses were associated together to the exclusion of laymen. The result shows that this idea was ludicrously impractical, for it has led to discord, and revealed a conspicuous absence of business management and method. No doubt a majority of the R.B.N.A. will learn business by experience. If this indeed be the case we shall soon see the introduction of business methods and the exfoliation of the small and truculent section which has caused all the mischief. When these necessary changes have been effected the R.B.N.A. may hope to enter upon a peaceful and useful career full of benefit to those for whose protection it ought to exist and flourish.

### GOOD HONORARY SERVICE.

The committee of the South Wales Nursing Institute are losing the valued services of Miss Aubrey, who is resigning the office of hon. secretary to the institute, which she has held for the past twelve years, in fact, ever since its formation. At the annual meeting the best thanks of the subscribers were cordially voted "for the kind and highly efficient manner in which she had devoted herself to the interests of the establishment from its commencement until the present time, together with their cordial wishes for her future health and happiness." Miss Aubrey has been elected a member of the committee.

### NURSING AT THE STOCKPORT INFIRMARY.

The nursing staff at the Stockport Infirmary has been recently increased. There are now two sisters and ten nurses and probationers, under the superintendence of a thoroughly-trained matron. The probationers serve for a term of three years. Courses of weekly lectures are given by the house surgeon, which are much appreciated by the nurses. We trust the increase in staff has been coincident with improved accommodation for the nurses, for this was very inadequate a short time ago, and the available space quite disproportionate to the demand upon it.

### A STEP IN THE RIGHT DIRECTION.

It is very satisfactory to learn that the authorities of the Savernake Hospital have found the increase in the nursing staff so successful in every way. When a visit was paid to the little institution some time ago, we felt that a weakness in the management was the inadequacy of the nursing arrangements, one nurse only being in charge both for night and day. Now the matron has two fully-trained assistants. The nurses will find quite enough to do with attendance on from eighteen to twenty patients, but the staff now working at Savernake may be regarded as fully adequate, and as setting a good example to many less well-managed cottage hospitals throughout the country.

### WEST BROMWICH GUARDIANS AND THE LOCAL GOVERNMENT BOARD.

The West Bromwich Board of Guardians have recently had occasion to fill the post of head nurse at their workhouse infirmary, vacant by the resignation of Miss Carpenter, who during the last nine or ten years did much to raise the standard of nursing in the infirmary. The Guardians, it appears, in advertising for her successor, stated that applicants must be "hospital-trained and certificated nurses, between twenty-five and forty years of age." Certain suitable candidates were duly selected by the House Committee to appear before the Board, who, however, finally appointed one of the under-nurses in the infirmary—a woman of fifty-five, with no hospital training. This decision the Local Government Board have very rightly refused to sanction. At a meeting of the Board held last week, Mr. Longe, one of the Guardians, pointed out that present-day views with regard to the importance of nursing in workhouse infirmaries had to be considered, and that they were appointing a person of considerable age, "who had never had any hospital training, and who had simply filled the position for several years of an under official, to the position of head nurse to an infirmary of 220 patients, and this without taking the opinion of the medical officer on the matter." Several members of the Board held Mr. Longe's views, and advocated the setting aside of the appointment, but after some discussion the Chairman moved, and carried by one vote, a resolution that the "clerk write to the Local Government Board strongly protesting against their action, and requesting them to consider their decision." It is to be hoped that the Local Government Board will remain firm and will insist upon a properly trained and certificated person being placed in the responsible position of head nurse. That post at Bromwich is one requiring exceptional ability and experience, for we understand that there are sometimes as many as 270 patients in the infirmary, that these include acute and surgical as well as chronic cases, and that there is no resident medical officer. Local opinion in West Bromwich is strongly against the action of the Guardians, and from letters we have received we gather that much indignation is felt in the neighbourhood at their failure to realise the responsibility which rests with them to provide adequate superintendence over the sick under their care.

### BANGOR TRAINED NURSES' INSTITUTION.

Another year's "prosperous and increasing work" is reported by this institution, which has lately added to its outlying districts that of Tregarth, and now employs eight district nurses, of whom six are Queen's nurses; 5,234 visits were paid during 1895. There are ten nurses on the private staff, who attended one hundred and four cases during the year. Dr. Langford Jones, speaking at the annual meeting the other day, spoke in appreciative terms of the work done by the institution and commented on the different state of things which had prevailed not so many years ago, when "the theme of the medical men's speeches had been the prejudice nurses had to contend with in entering the homes of the poor people." Now the difficulty was to cope with the demands upon the nurses' services, which were valued more and more each year. Lady Penrhyn is the energetic president of the Bangor institution, in which she takes a keen personal interest, and its valued lady superintendent is Miss Vickers.

### NEW BUILDINGS AT PORTSMOUTH.

The foundation-stone of a hospital and nurses' home was laid early this month by the chairman of the Portsea Island Board of Guardians, the new buildings being supplemental to the present infirmary, and the two pavilions of which they will consist occupying an additional four acres. The administrative block and nurses' home will suffice for the whole infirmary, and will comprise on the ground floor rooms for the medical officer and the matron, with the nurses' dining-rooms and night attendants' room connected by telephone with the wards; on the first floor separate bed-rooms for the nursing staff and their sitting-room, while the top floor is to be devoted to the servants.

### THE POISON BOTTLE AGAIN!

An unfortunate accident has occurred at the Birmingham Workhouse Infirmary, a man having died from the effects of an injection of morphia intended for another patient, administered by the charge nurse of the ward in error for the solution of strychnine ordered. The mistake was discovered at once, and every means tried to avert the effects, but the man died two hours later. At the inquest it was stated that the post-mortem examination revealed the fact that the lungs and heart were in an irremediable condition, and that morphia had no doubt accelerated death. The jury added two riders to their verdict of "death from misadventure," to the effect that as far as practicable all subcutaneous injections should be given under the superintendence of the resident medical officers, and urging that it should be rendered impossible for the nurses to obtain or administer any injection other than that prescribed for the patient. And here is where the moral comes in; for the nurse (who was entirely acquitted of blame by the coroner) stated that "the morphia and strychnine bottles were alike in style and colour and form of label. The cupboard, which contained about forty such bottles, had been cleaned out, and the bottles must have been disarranged." It is certain that when a number of bottles containing poisonous drugs are kept together there should be some method adopted by which they shall be easily distinguishable one from the other; and also the oft-repeated warning to every nurse to "read the label" at all times before the administration of drugs, no matter how impossible any mistake may seem, must be emphasised once more.

### SHORT ITEMS.

Anonymous letters are usually disregarded, but the following one, dated Wolverhampton, March 25th, may probably be published: "Please say in your next book called The Hospital if it required an educated person for hospital nursing." Uncertain.—The Gloucester Board of Guardians have rescinded a resolution passed by them ten years ago, and have determined, in the face of the present epidemic of small-pox, by 31 votes to 22, to put the compulsory clauses of the Vaccination Act into practice.—Efforts are being made, and apparently successfully, at Buntingford to start a district nurse for that place and surrounding parishes. It is intended to affiliate with the Jubilee Institute. A committee has been appointed to receive subscriptions.—Lady Victoria Lambton read an interesting paper on "Rural District Nursing" at the sixth annual meeting of the Welsh branch of the Queen's Jubilee Institute, recently held at Cardiff.—At the recent annual meeting of the Burton Nursing Institution the Mayor, Mr. J. R. Morris, who presided thereat, promised a donation of £10 to the institution if five similar sums were forthcoming, in order to clear off the present deficit of £49 on last year's working.—The annual meeting of the Dufferin Fund was held in Calcutta on March 13th, the Viceroy presiding.—Miss Adèle Isabella de Steiger, M.B.Lond., has been appointed third medical officer to the Essex County Lunatic Asylum, Brentwood. Certain alterations have been made in the rules of the institution to make the election of a woman to this post in order.—Lady Halsbury has handed over to St. Michael's Convalescent Home, Westgate-on-Sea, the substantial sum of £530 17s. 6d. towards the £1,000 required for the building of the new wing, the result of the ball recently held at the Inner Temple Hall in aid of the charity.

### On Certain Aspects of the Nursing Question as Seen in England and Germany.

By a Certificated Midwife.

V.—THE HEBAMMEN SCHULE—(*Continued*).

I promised to say more about the manual of instruction which was in use at Stuttgart, and which we were all obliged to know thoroughly or our certificates were not forthcoming. The one that lies before me now is the one used at Dresden, but as the books follow the same system in all the schools that I have visited it will make no difference which I describe. On the title page are the words, "Commanded by the Royal Saxon Ministry of the Interior." It is written in the most simple and terse manner, the terminology throughout being couched in the vernacular. In this respect German pupils have an immense advantage over ours; as in English it is the custom to use only words derived from Latin or Greek roots in describing the mechanism or processes of childbirth. Thus, women who have had no classical education are obliged to learn by rote a new vocabulary, which, when learnt, conveys no living idea to the mind. To give an instance: I open my manual at random and light on the word *Zwerchfell*, which explains itself either to a native or one who has learnt German. Our English word is *diaphragm*, and where is the pupil in any of our lying-in hospitals who knows the meaning of that by the light of nature? Again, look at this term *sacro-iliac synchondrosis*, and imagine if you can the average intellect of an ex-ladies' maid struggling with it; and then turn to the German peasant woman who, when her teacher puts his finger on the particular joint in the skeleton indicated by that horrible word and asks, "What do you call this?" replies, out of her own head, "*Hüft-bein-fuge*."

These are no exceptional instances, but such as are to be found on every page. Thus, with less definite instruction given, less time allowed for learning, and much less opportunity of learning the English pupil has a grievous burden imposed on her powers of memory as well.

The *Lehrbuch* begins, as I have said before, with the framework of the human body, and goes on to its organs and functions. After that it proceeds to teach the process of normal childbirth in all its details, going on to abnormal cases and explaining their danger or difficulty, at the same time laying down strict rules as to what an "*angestellte*" or Government appointed midwife may undertake, and what is forbidden her to attempt. At the end of the book come some of the most common diseases of women and infants, and how to recognise and act with regard to them. Finally there are various simple remedies explained, and the book winds up with the "duties and obligations of the midwife," and the oath which she has to take. As this oath is the sign and seal of the difference between the two nations in the way they regard the profession of a midwife, I will translate it in full:—

"I (M. or N.) swear herewith before God the Almighty and Omniscient, that I will ever follow truly the well-known precepts and rules of my calling; and will carry out faithfully and exactly all that it behoves an honest midwife to do. So may God and His Holy Word help me."

The difference of view lies in a nutshell. In one country the calling is a vulgar one, in the other it is holy. In one all the instruction given about it must be wrapped up in a terminology, "not understanded of the people," to save offence to ears polite; in the other, nature and the language of the people are invested with a new dignity by the authority of the State. And I leave my readers to form their own conclusions as to which view is to be preferred.

Meanwhile let me describe the house or hospital at Stuttgart before I go on to the others I visited. It stood in the outskirts of the town in the midst of a shady garden, where the pupils and patients might often be seen sitting under the trees. On the ground floor lived the housemaster and his family, and the patients who were not yet confined. The number of these, of course, varied from day to day, but we may take twenty as an average. They did the work of the house under direction—washing, cooking, cleaning, &c. On the first floor came the room of the chief resident midwife, a large labour ward, and two or three for the mothers and infants. These were all front rooms and sunny. The back ones were given to the pupils.

On the second floor reigned the under resident, and the division of rooms was similar. At the top of the house was the theatre, and the apartments of the house surgeon and our chief, Herr Dr. Fehling, who, however, did not sleep in the building, though he had a consulting-room there. This was, as far as I remember, the whole of the building and its working staff. There may have been an occasional "*Tagfrau*" or charwoman had in for cleaning, but there were no staff wardswomen or night nurses. The pupils and patients did the work between them, overseen by the housemaster and his wife downstairs, and by Fräulein Scholastica and Fräulein Marie upstairs. The former always visited the women downstairs early in the morning to decide who was in a condition to work, and who would be wanted upstairs for her class.

The housemaster or steward was an elderly man, being fatherly in his bearing to us all. He bought in all stores, and kept account of all outgoings and incomings both of pupils and patients. He had the bearing of an old soldier, and his discipline was exact. Under his rule everything was in place, everything decorous, and no unseemly laughing or tattling was ever heard. The house surgeon, as with us, came and went with each term or each year, holding his appointment as a means of gaining experience. With him we had but little to do.

The fees charged for the training are very small, but I cannot remember the exact sum. I know what I paid myself (but I was an *Ausländerin*) was equivalent to about 30s. for the term. This was considered a large sum, as I provided my own board, and had a room across the road to pay for. But it was a charge made by the State, and not by the hospital, which received absolutely no pecuniary benefit from my admission as a pupil. In my native fashion I tried on leaving to make some little tokens of gratitude to the staff for all the kindness which had been shown me, and the privileges accorded to me as a foreigner entirely without claim to any; and I blush even after nearly twenty years when I think of the snubbing I received. "We are paid by the State," was the attitude they took, one and all; "We are pleased to have been allowed to teach you, and glad you have profited by our instructions, but for the rest — " and nothing more was possible than an affectionate and grateful good-bye.

One last word before I finish my account of the hospital must be permitted to me, since it is the key to the whole situation and my apology to the public for writing these articles.

"Herr Doctor," said I, cogitating on the fact that no death had taken place since my arrival, "what is your death rate?" "Death rate?" said he "we have none; why should the women die? Childbirth is not a disease."

### A Private Case in the Country.

An urgent summons to an amputation case, and I was whirled away to a neighbouring town where an uncompromising-looking cart awaited me, in which was seated a buxom farmer's wife. In a state of mind bordering on apprehensiveness, I mounted the aforesaid vehicle, and was jolted along rough country roads to a hamlet ten miles distant, thinking sadly of easy springs, cushions, and rugs, of which only a limited supply of the latter had been provided. During our long drive my charioteer informed me of the serious illness of her father, the impending operation, and the lonely situation of my surroundings for the next few weeks. A private nurse has queer experiences in the course of her professional duties, and truly my present case bid fair to be very peculiar. Picture the best kitchen of the farmhouse, extremely primitive arrangements in the way of operating table, basins, &c., a very old-fashioned fireplace filled with blazings logs, an ancient dresser adorned with old china, &c., a huge sofa destined to receive the recumbent form of the nurse during long wakeful nights in the near future, a bed in one corner for the patient, and the presence of four doctors to sanction the proceedings. I speedily donned cap and apron, and without waiting to drink a cup of tea invitingly offered, presented myself at the scene of action. The *modus operandi* of the surgeon was speedy and skilful, and within two hours the old farmer was comfortably fixed in bed, and the doctors preparing to leave. It must be confessed I felt some inward trepidation as I realised that I was in charge of a serious amputation case, three miles and a half away from medical assistance. However, I hoped for the best, and nothing more serious than oozing through the dressings occurred before the welcome evening visit of the nearest doctor. During the night my patient was extremely fractious, wondered what was the use of a nurse if she would not remove the bandages, and in the Devonshire vernacular assured me, "it's a pracking and a' smurting like mad itself." All my powers of persuasion failed to convince the old farmer that all was going on well, but at last the long night was over, and with considerable relief I mounted the stairs to seek a little repose, leaving minute instructions to a local Sairey Gamp, who was to keep guard in my absence. My room was comfortless and dirty, and, indeed, the sanitary arrangements of the entire household left much to be desired. I slept for an hour or two, and then hurriedly prepared for the doctor's arrival. My patient rebelled strongly against "rummage" and "tackle," commonly called beef-tea and milk; wondered why I took his temperature, grumbled incessantly, being quite sure he would never recover, prophesied a most dismal future as to the prospects of the farm produce, and did all in his power to hinder his recovery; but in spite of everything the leg progressed most satisfactorily. The daily visit of the doctor helped to sooth my ruffled feelings, and was, indeed, almost the only link connecting me with the outside world, for the house was situated in a very lonely spot, miles away from any church or post office, and literature of any kind was very scarce indeed. The old farmer protested strongly against the processes of ablution, was indignant at the idea of my going to church, wondered why I did not take my meals at the same table as the farm labourers—who dined with the family in the kitchen—and continually demanded his customary "taters and bacon" and bread and cheese. The daughter-in-law of the old man did her best to compensate me under the circumstances. She sent me out in a jolting cart to get some fresh air, and so thankful was I for a change of scene, that the old cart seemed a luxurious conveyance. I was compelled to borrow an old pair of extra thick boots from the good woman for my journeyings across the farmyard in muddy weather, for though the house stood upon very high ground there was a great deal of clinging mud, which filled me with dismay every time I ventured outside the premises. Before many days had passed the only domestic of the establishment went home ill, and I was reduced to a daily sweeping, dusting, and grate-tidying of my patient's room, to say nothing of constant journeys to the back regions in search of wood to keep up the fire, there being no coal kept on the farm premises. The wonderful energy of that housewife was worth seeing. She rose early, prepared all the meals, did all her own dairy work, made bread, sewed for the family, fed and attended to her poultry, and was always cheerful. The one thorn in her side was her discontented old father-in-law, whom nothing could please. I was thankful to be able to lighten her burdens somewhat, and joyfully undertook a bundle of needlework, as my patient improved rapidly, and did not need constant attention. I must not omit the bright side of the life I led in that lonely farmhouse, for in spite of much that was discouraging there was a bright side. Glorious views of the surrounding country could be obtained from the neighbouring fields. Rich tracts of land were under cultivation in every direction, and that peculiar purity of atmosphere which is only seen in regions remote "from the madding crowd" enabled me to see objects at a great distance with comparative ease. The air was pure and bracing, and I was able after a long lonely tramp to do full justice to the rich Devonshire cream, new-laid eggs, and home-made bread provided by my excellent friend, the aforesaid mistress of the farm. The first Sunday after my arrival, after surmounting a few difficulties, I was driven to a neighbouring village with the intention of attending a service in the parish church, but, alas for my hopes, the parson had taken cold and could not preach, and our long cold drive was all in vain. The next Sunday found me seated again in the family cart, holding a huge green umbrella with fingers benumbed with the cold, being driven to church in a severe hailstorm by a small farm boy. On this occasion the service did take place, and I was glad to be once more within "sound of the church-going bell." At length, to my complete satisfaction, the doctor pronounced my patient well enough to be left to the care of the Sairey Gamp before-mentioned, and I took my departure in a highly-respectable gig, the property of a butcher a few miles away. The long drive in the bracing air of a March morning restored my drooping spirits, and I was able to laugh heartily as I retailed my experiences a few hours later to a sympathising circle of friends. Upon mature consideration I am glad to have had a glimpse of the kind of life some of our English farmers lead—a life in which books, newspapers, pictures, lectures, and ordinary social intercourse have no part, and in which, as a rule, no event more startling than a visit from a neighbouring parson or a weekly jolt to market varies the monotony of the colourless days from January to December; but *chacun à son goût*.

### Minor Appointments.

St. Thomas's Hospital, S.E.—Miss Allardyce, recently Sister Clayton, has been appointed sister of the new Florence ward at St. Thomas's Hospital, the charge of Beatrice ward having been given to Miss Easton, late Sister Elizabeth. Miss Garvey and Miss Tippett have been respectively promoted to the wards left vacant by these changes.

### Midwifery Papers.

### II.—CHANGES IN PREGNANT UTERUS. PLACENTA—FŒTAL HEAD.

The changes in the uterus after conception has taken place are very marked. Dr. Playfair has said, in his book on midwifery, that the modifications which occur in the transformation of the small non-pregnant uterus into the large fully-developed uterus of pregnancy are most marvellous, and "have no parallel in the whole human economy." In order to render the pregnant uterus strong enough for the work it has to do, its muscular walls become thickened, while, at the same time, they grow softer and more elastic; the mucous membrane, lining the walls internally, is greatly thickened, and there is much distension of the blood-vessels and lymphatics. There is special enlargement of the blood-vessels at the fundus, over what is known as the placental site. The cervix, or neck, of the uterus secretes an increased quantity of thick white mucus; it becomes much softened, and turns a dark violet colour owing to the congested blood-vessels and the effusion of serum in its substance. The pregnant uterus at "term" (or end of pregnancy) weighs 2 lbs., and is 12 inches long and 9 inches broad.

When the impregnated ovum escapes from the Fallopian tube into the uterus, it is received and retained at the fundus by the thickened mucous membrane, which blocks up the uterine cavity. This thickened membrane is called the decidua, and is split into two portions, one—the decidua vera—lines the uterine cavity throughout, the other—the decidua reflexa—is closely wrapped round the ovum. That part of the decidua vera on which the ovum rests, and from which the placenta eventually develops, is called the decidua serotina.

The placenta is the important organ which conveys nourishment and oxygenation from the blood of the mother to that of the fœtus (child), and its substance is full of blood-vessels which are closely associated with those of the mother at the placental site. It is about 6 to 8 inches in diameter, and weighs from 18 to 20 ounces. On its maternal surface (the side which is attached to the fundus) it is rough and broken up; the fœtal side is smooth and covered by membrane. The umbilical cord grows near the centre of the placenta on the fœtal side, and is attached by its other extremity to the abdomen of the child. It is about 21 inches long, is white and glistening, and encloses the artery and vein which convey the blood between the placenta and fœtus. After the birth of the child, but before the expulsion of the placenta, this artery can be seen swollen and pulsating through the white membrane of the cord.

From the placenta grow the three membranes which form the bag or sac in which the fœtus is enclosed. They are the amnion, the chorion, and the decidua reflexa. The amnion is the internal membrane, and immediately encloses the liquor amnii in which the fœtus floats and grows. The chorion lies between the amnion and tho decidua reflexa, which has already been noticed. These membranes are attached to and are expelled with the placenta, with which they must be carefully examined after birth to see that they are complete, and that no pieces or shreds are left behind in the uterus to set up secondary hæmorrhage. The three membranes can be easily distinguished in examination, and can be peeled away from each other. The liquor amnii is a most useful provision of nature for the protection of the fœtus, which is suspended in it, from the effect of (1) shocks or jars to which it may be subjected from accidents to the mother, and (2) from the strong pressure of the uterine walls. It also prevents injury to the uterine walls by its gentle and slowly increasing pressure aiding gradual distension. During labour it lubricates the passages and thus assists the descent of the child, but its chief use is in forming with the membranes, soon after the onset of labour, a fluid wedge for the dilatation of the os-uteri. (This action will be further explained in a note on the mechanism of labour.) The liquor amnii has a slight odour, and is generally clear and limpid in appearance. Occasionally it is tinged with green, owing to the presence of meconium, which has been excreted by the fœtus before the membranes were torn. Offensive and dark-coloured liquor amnii often indicates some abnormal condition on the part of the mother or child.

The fœtus at term weighs about 6 lbs. to 8 lbs., and its length is about 18 inches to 21 inches. It is generally covered at birth by a white, cheesy material called the vernix caseosa. The bones of the fœtus are very soft and gelatinous, and easily compressible. The head, owing to its relative heaviness, is generally the "presenting part" (the part which descends in the pelvis and is generally born first) in labour, and it is necessary for the midwife to recognise and localise the bones composing it.

The fœtal skull is formed of seven bones—two parietal, two frontal, two temporal, and the occiput. The parietal bones are situated one on each side of the upper part of the head, and form the arch of the skull; the temporal are placed on each side of the skull towards the front and above the ears, and the frontal bones form the forehead. The occiput is placed at the back, and form the lowest part of the skull. All these bones are soft and yielding, and overlap one another when the head is passing through the pelvis. They are joined by membranes and the lines of union are called sutures; the membrane-covered spaces lying between the sutures are called fontanelles.

The four principal sutures to remember are (1) the frontal dividing the forehead bones; (2) the sagittal, a continuation of the frontal running between the parietal bones; (3) the coronal running across the head, separating the parietal and frontal bones; (4) the lambdoidal dividing the occiput from the parietal bones. The fontanelles are (1) the anterior or greater is the interstice between the two halves of frontal bones and the two parietal. It receives four sutures, the frontal, the coronal (one on each side), and the sagittal. (2) The posterior or lesser fontanelle lies between the point of the upper edge of the occiput and the two points of the upper border of the parietal bones on each side. It receives three sutures, two lambdoidal and the sagittal. The recognition, by vaginal examination, of the position of the fontanelles and the sutures running into them, is a great help in diagnosing the "position" or way in which the head will be born, also in ascertaining by their change or non-change of position the progress the labour is making.

### The Nurses' Co-operation.

The Nurses' Co-operation is to be heartily congratulated upon the excellent appearance of their report and the prospectus which they have just issued, which contains a record of the five years' work of the institution. We have previously referred to the result of the annual meeting, and it is not therefore necessary to dwell again upon the wonderful progress which has marked the movement. Our object is to bring the two dainty little pamphlets to the notice of our readers, and especially to those who are occupying themselves in producing similar literature. The cover of the prospectus is especially attractive. It is formed of a pretty grey-blue linen, slightly stiffened, lettered in black, and relieved by a facsimile badge in red and white. It contains, besides the report of five years' progress, the names of all officials and nurses connected with the co-operation, and a list of fees. The report gives the work of the past year, together with a financial statement and the rules and regulations of the institution. The whole is admirably got up.

### Talks About Food.

### VIII.—AN "EXCELLENT SUBSTITUTE."

We have already spoken of the strong predilection of our poor people for white bread and tea—a predilection which has doubtless been greatly fostered by the tremendous fall in the price of bread, brought about by the repeal of the Corn Laws and by successive reductions in the duty on tea. No one, of course—unless it be the farmers—can regret that an important article of diet like bread should be procurable at so low a price, but even so good a thing as cheap bread has, it is to be feared, *les défauts de ses qualités*; and those defects are really serious if they result in keeping the poor on one monotonous diet. It is rather a startling fact—if it be a fact, as millers declare—that when flour is dear, more of it is used than when it is cheap. The suggested explanation is that, when flour is dear, poor people become aware that they will have but little money to spare for what may be termed luxuries, such as meat and butter, and, giving up these, eat more bread. But if this be so, it shows a lamentable want of resource in the matter of diet, and of knowledge of the excellent foods which might well supply the place, in part, at any rate, of the wheaten loaf now thought so indispensable.

It is a thousand pities that we cannot get English people to take kindly to that mainstay of the Scotch labourer, oatmeal. But, indeed, we doubt if the effort has ever been seriously made. It is no easy thing, in the country, at any rate, to get good oatmeal. Very often it is not kept at the village shop; or if it is, it has been in stock so long that it is fusty and tasteless, and has quite lost the pleasant smell which it has when fresh. Ladies Bountiful might do a good deed by showing the village people how to make porridge, and by then taking care that good oatmeal should be available, getting the village shopman, or some other person in the village, to keep it. We cannot believe but what such efforts would be crowned with success—in time; we say *in time*, because country people are proverbially slow to make a change. But it almost stands to reason that children will prefer a hot breakfast of porridge to the hunch of bread, with or without "scrape," and the draught of (very often cold) tea, which generally forms their morning meal. Now and again you may find a child who does not like the taste of oatmeal, or upon whose sensitive mucous membrane its roughness has an irritating effect; but as a rule porridge will be found both pleasant and wholesome if only it is properly cooked. Doubtless we must expect a little difficulty about this at first; but, after all, porridge-boiling is by no means as difficult as bread-making, an art in which almost every cottagewoman is proficient; and if she can learn the one, she can learn the other.

We do not know whether porridge-making is part of the course of cookery now taught in our Board schools; if it is not, it ought to be. While oats contain nearly as much nitrogenous matter as does the kind of wheat generally used for bread, they contain a much larger amount of fatty matter, and are also rich in mineral matter, being thus a valuable bone-making food. And when we consider, further, that flour is much inferior to wheat, being robbed by the miller of a great part of the nitrogenous and mineral matter which it ought to possess, we see at once the high comparative value of oatmeal.

Maize, again, is an excellent food, especially for cold weather, containing by far the largest amount of fatty matter of all our cereals; and we might well follow the example of our American cousins, who prepare it for the table in numerous ways, as may be seen by their cookery books.

We must remember, however, that it is not porridge merely which makes the Scotch labourer strong and hearty, but porridge and milk. The importance of milk as an article of diet has already been urged in another article, so it only remains for us to say here that milk should always be spoken of as the natural adjunct of porridge, and that thus, if we can persuade the poor to take to porridge eating, we shall be killing two birds with one stone.

Another thing which should be impressed upon them is the importance of regular meals. Many poor children (and mothers, too, for the matter of that) hardly know what it is to sit down to a meal; or if they do, it is only once in the day. As a general rule, they ask for "a piece" when they feel to want it, the result being that the little stomachs are never fully satisfied and never at rest. As Miss Tabor graphically puts it (we quote again from *Labour and Life of the People*), "They (the lower class of London school children) have no regular meal-times. When they are hungry the mother puts into their hands a 'butty,' *i.e.*, a slice of bread with a scrape of dripping, lard, or the current substitute for butter, and sends them off to consume it on the doorstep or in the street. The youngest of the brood she supplies with a 'sugar butty,' *i.e.*, a butty with as much sugar as will stick upon the scrape. A draught of stale tea usually goes with it. When funds are low, or when drink forestalls the children's bread, the scrape and cold tea vanish, the sugar butty is a thing of the past, and the slice from the loaf becomes an intermittent supply; neighbours help out the children's needs, and free meals at school keep starvation from the door."

It is much the same with country children; and the evil effects of such a system of feeding, both upon the children's physical organisation and upon their manners, are very evident. Mothers of families should be urged to provide meals at regular times, to insist that the children shall sit down to the table and eat what they want, and to refuse to give anything, except in special circumstances, between meals. The provision of porridge, or of anything that needs to be eaten hot, will be a great assistance to her in forming this habit. At present "a man who has his three meals reg'lar" is, in some parts of the country, a synonym for a man who is thoroughly well-to-do; but there is no reason why working people, who usually have regular working hours, or at any rate, their children, whose school hours are like the laws of the Medes and Persians, should not in their humble way emulate the well-to-do man's example, and at least have their two meals reg'lar.

### Appointments.

Brook Fever Hospitals, Shooter's Hill. — Miss Emmeline Mary Bann has been appointed matron of the above hospital. She was trained at the Royal Infirmary, Derby, where she held the post of charge nurse for two years. She subsequently acted as night superintendent at the Worcester and Gloucester Infirmaries, and undertook private nursing for a short time. She has been matron at the Hull Sanatorium for four years, and leaves there for Shooter's Hill. Miss Bann's testimonials are excellent throughout her career, and we wish her equal success in her new position.

Convalescent Home, Bearsden.—Miss Sophia Monro has been appointed to the matronship of this new convalescent home, given to the Royal Infirmary, Glasgow, by Miss Schaw in memory of her brother, the late Mr. Archibald Shanks Schaw. Miss Monro was trained at St. Mary's Hospital, Paddington.

Cheltenham General Hospital.—Miss Howes has been appointed matron of this hospital. Miss Howes received her training at St. Thomas's, at which hospital she has held the posts of ward sister and night superintendent, only now leaving to take up her new appointment at Cheltenham, accompanied by many good wishes from her fellow-workers.

Walton Convalescent Home.—Miss Edith Sutcliffe has been appointed matron of the Convalescent Home at Walton-on-Thames. She was trained at St. Mary's Hospital, Paddington, and subsequently held the post of matron at the Hospital for Women and Children, Leeds, and at the Royal Bath Hospital and Rawson Convalescent Home, Harrogate. Miss Sutcliffe enters upon her duties at the end of April.

### Nursing in Crown Colonies.

By Walter Maguire.

### BRITISH GUIANA.

I am aware of the many objections which may be urged against any attempt to improve, or any interference with, the condition of affairs, described by me as at present existing. First must be considered the bad financial state of the sugar plantations—many having been completely abandoned with machinery and plant years ago—a state which has grown worse every year, and which, through the introduction of beetroot sugar so near home, has crippled the resources of many large planters; again, it will be urged that attempts have before been made to ameliorate the condition of the coolies (and to which your previous correspondent correctly refers) by the nursing and medical profession. Some years ago a lady, the daughter of a planter in Essiquibo, and well known for her philanthropic principles, attempted to establish a home or institute of visiting nurses in Georgetown, but failed (not, however, till her private resources were exhausted and her health destroyed by hard work and bitter disappointment) for want of support. The managers of the various estates could hardly be expected to contribute largely out of a salary, in many cases, barely sufficient for the conditions which their positions demanded, and the proprietors of nearly half the plantations in the colony reside in Europe. The residents of the towns—principally merchants and shop or store keepers (a fourth of whom are Chinese or Portuguese, whose reputation for Christian charity is not proverbial)—are quite disinterested; and, further than chance opportunities arising from their business transactions or periodical visits by the estates people to their stores may enlighten them, they know very little, and care less about plantation life.

It is greatly to be feared also that the custom of "looking sideways" on the "nigger" is as essential a characteristic of the average colonist of British Guiana as in the days when the old Dutch taskmasters (by methods of persuasion so approved by them and so little appreciated by their "blackbirds") reclaimed from sea and savannah water this vast tract of malarial coast-line from the Orinoco to the mouth of the Maroni River.

Be this as it may, the callous indifference and apathy with which all matters affecting the interests of this vast Indian emigrant population are regarded, and the marked absence of the softening influence which modern civilisation is calculated to produce, demand the serious attention of all who are interested in charity and progress.

Now first I would suggest that a committee composed of ladies interested in nursing be formed in London with a gentleman of the medical profession as president. That a fund be started, and the signatures of the leading proprietors of British Guiana plantations be secured as subscribers, as Messrs. Ewing and Co., of Glasgow, for instance, followed by appeal to the Press for assistance. The *bonâ fide* sincerity of the undertaking would be guaranteed by the position of its president. That a deputation be sent to Georgetown, and there establish a centre of operations and add to their numbers. They would enlist the sympathy and support of many who, although too faint of heart to pioneer the movement, would willingly enter its ranks.

Operations could be commenced by visiting the "nigger yards" of the estates after work for the day in the fields was done, and the work of visiting could extend to Berbice (with a small depot in the town) and the villages and estates around it, as far as plantation "Sheldon," and others on the Corentin coast. The overseers' quarters would also come in for a share of attention, sadly needed. These young fellows, many mere boys, fresh from Europe, are continual sufferers from the malarial fever which an overseer's life, and exposure to heavy rains, and the poisonous atmosphere of the swamp induces; but in their restless tossings they can receive no sympathy or the soothing comfort which only a woman knows how to bestow. Who in Berbice among the old overseers does not remember dear old Mother Welchman, the mother of the manager of "Blairmont," and her daily visits (nearly till her death) to her "son's boys"?

The medical men of the colony are powerless to act. Their work is already too hard, and the remuneration small. No; the only remedy lies in the pockets of the proprietors. Here, then, is an opportunity for many who must find the field of workers here getting very full.

### The Society of Chartered Nurses.

We have received particulars of the constitution and conditions of membership of "The Society of Chartered Nurses, a Co-operative Society of Members of the Royal British Nurses' Association," which has been organised, and offices started, at 24, Princes Street, Cavendish Square, W. "This society," the prospectus states, "has been instituted with the primary object of giving greater opportunities for obtaining employment to members of the Royal British Nurses' Association." It is conducted on co-operative principles, and the constitution is as follows: (1) The management is vested in a committee of medical men and matrons. (2) Each nurse will receive the full amount of her earnings, less a deduction of $7\frac{1}{2}$ per cent., which is placed to a maintenance fund, from which the working expenses of the society will be defrayed. The accounts of the society are audited annually by a chartered accountant. Any surplus which may then remain, together with receipts from other sources, will be placed to a reserve fund. (3) The committee is a purely honorary body, and the rules, which are, or may be, framed, are for the advantage of the members, who, it is hoped, will remember that the professional and public standing of the society will, to a large extent, depend upon their loyal co-operation. Candidates for membership of the society must be members of the Royal British Nurses' Association who have had three years' hospital training, and all selected candidates will be engaged on three months' probation before being finally elected to membership. Sir James Crichton Browne and other members of the executive of the Royal British Nurses' Association are on the committee.

### Novelties for Nurses.

### THE LONDON SHOE COMPANY.

Owing to the notice which appeared in our columns of the recent sale at the London Shoe Company, such a number of ladies attended, that many nurses, amongst others, went away disappointed. The manager assures us that the next sale will be more conveniently conducted at their new warehouses in Queen Victoria Street, E.C.

### Medals at the Cookery Exhibition.

A special gold medal is offered by the Baroness Burdett-Coutts at the Cookery and Food Exhibition, which is to be held at the Imperial Institute from April 27th to May 2nd, for the best set of dishes suitable for invalid cookery. Other prizes for invalid and sick-room cookery are offered by Mr. Pearson, of *Pearson's Weekly*, and Miss M. Tattersall.

### Practical Points.

*Female Nurses and Male Lock Cases.*—"*Infirmary Nurse*" *writes: Will you kindly inform me, through your column for "Notes and Queries," whether, in your opinion, a female nurse should nurse male lock cases? I am a staff nurse at the above asylum, having a medical ward of thirty-two patients, in which a certain number of beds are reserved for these cases. Not only myself, but several of the better-class patients in my ward, object to this arrangement. I have appealed to the Medical Superintendent and Matron, and they tell me they can do nothing in the matter for an indefinite period. As the present arrangement has been in existence for a number of years, they appear to see no ground for complaint. Not wishing to resign my appointment on this account, I am anxious to know if I can take any further steps in the matter.*

[The particular case mentioned by our correspondent proves that lock patients should not be mixed up with other cases in general wards. All such cases should be classified and treated in separate wards, the male patients being in charge of male nurses wherever possible. In regard to places, however, where this is impossible, the question put to us is a difficult one to answer, and it is quite impossible to give to it a simple "yes" or "no." In the first place, there are nurses young and nurses old, and what might apply to one need not of necessity apply to all; then there are nurses delicate, beautiful, and to male eyes attractive, and, we dare say, some are to be found who are just the opposite, so that no rule can apply generally. Our first impulse, however, on reading the question which has been put to us, is to answer definitely "No," and in regard to many nurses we should adhere to that answer. There is, however, another side of the question which cannot be shut out of sight. We have to consider the sort of things which medical women feel themselves qualified to undertake, and in some instances (wrongly, as we think) take a pride in nerving themselves to do; and, on the other hand, the sort of things which are accepted without hesitation as part of the daily work of nurses in general and fever hospitals. A nurse will without hesitation undertake the nursing of a case of suprapubic cystotomy or lateral lithotomy; she will attend to a case of operation for fistula or hæmorrhoids, and will as conscientiously keep the wounds in a cleanly condition as if the operation had been upon the scalp; through the long weary days of a typhoid case she will attend to a patient in a way which, however necessary, is one which would be revolting but for the fact that it is her work, her professional duty, and that other considerations really never enter her head. The fact is that every day of her life a nurse in a large hospital has to do things which would be shocking and indecent if merely looked at from the conventional standpoint in regard to the proper relations of the sexes. The life both of a doctor in his dealing with women, and of a nurse in her dealing with men, would be abominable and disgusting were it not that such ideas never enter into their heads. Knowledge and duty must cover all these things, and both doctors and nurses must stick to that view which is expressed by the motto, "To the pure all things are pure," otherwise their lives would be degraded. In regard, then, to the question of lock cases, a nurse who has to look after them should dismiss from her mind all thought of the origin of such cases, and deal with them exactly as she would her other patients; that is, in regard to general nursing. In regard to local applications it is always the case that these things are done either by the patient himself, or by the medical officer. Any medical man will do his best to save a nurse from much that is disagreeable in such matters, and in fact the great objection to the appointment of women as assistant medical officers to workhouse infirmaries is the knowledge that in such an event whenever the principal medical officer was absent these cases would be thrown entirely into the hands of women. But that does not apply to ordinary nursing, in regard to which we can only repeat that the cause of the disease should not enter into the question. The amount that is done may well, however, depend on the ability of the patient to help himself. No nurse is called on to do what a patient can do equally well himself; and as to local dressing, that should fall to the doctor when it is more than the patient can manage.—Ed. *T. H.*]

### Everybody's Opinion.

**[Correspondence on all subjects is invited, but we cannot in any way be responsible for the opinions expressed by our correspondents. No communications can be entertained if the name and address of the correspondent is not given, or unless one side of the paper only be written on.]**

### PRIVATE NURSING BY THE DAY OR HOUR.

"Nurse A" writes: I was interested in the letter from "A Trained Nurse" under this head, in last week's Hospital. It has often occurred to me that an institution for sending nurses out to pay daily visits would fulfil a very useful purpose. I should like much to know if any nurses have tried the experiment on their own account, and how it has been received. I know myself of many a small and struggling household where in cases of long continued illness a nurse on these terms would be a most real help. If the need for such an arrangement is a real one, as I think it is, surely one or two enterprising nurses, with co-operation on the part of a few medical men, might start a scheme on this basis with a very fair prospect of success from a financial point of view.

### Notes and Queries.

The contents of the Editor's Letter-box have now reached such unwieldy proportions that it has become necessary to establish a hard and fast rule regarding Answers to Correspondents. In future, all questions requiring replies will continue to be answered in this column without any fee. If an answer is required by letter, a fee of half-a-crown must be enclosed with the note containing the enquiry. We are always pleased to help our numerous correspondents to the fullest extent, and we can trust them to sympathise in the overwhelming amount of writing which makes the new rules a necessity. Every communication must be accompanied by the writer's name and address, otherwise it will receive no attention.

### Queries.

*Nurse Alice* and *Nurse Mary* are reminded that full name and address must accompany all queries, or no reply can be given in this column. This condition is clearly stated above.

(1) *Training.*—Will you kindly tell me what to do? I have had twelve months' asylum experience, and for two years have nursed a private case (epilepsy and paralysis). I wish to continue nursing in some institution not an asylum. My age is 26.—*Nurse B.*

(2) *Temporary Work.*—Do you think it is possible to obtain work in any nursing institution or convalescent home for the summer months only?—*Sister Maud.*

(3) *Probationer.*—Can you tell me of any hospital where I could be taken as probationer? I am a domestic servant, twenty-one years of age.—*E. K. M. E.*

(4) *Lecturer (E.B.)*—Can you give me particulars as to examinations under the County Council, and training as a lecturer?

### Answers.

(1) *Training (Nurse B.).*—If you intend doing good nursing work your best plan will be at once to enter for a term of training in a general hospital or infirmary. Get "How to Become a Nurse" (Scientific Press, 428, Strand, London), and write to the matrons of the workhouse infirmaries mentioned on p. 73. You will also do well to watch the advertisements in The Hospital "Nursing Supplement," and reply to any that are suitable.

(2) *Temporary Work (Sister Maud).*—Your query is very vague, as you do not specify what sort of "work" you wish for or are able to take. You had better advertise and study the advertisements in The Hospital each week.

(3) *Probationer (E. K. M. E.).*—Get "How to Become a Nurse" (Scientific Press, 428, Strand, London, W.C.) and apply direct to the hospitals and infirmaries there mentioned, where probationers are taken as young as twenty-one. There are few, if any, general hospitals which do this, so if you do not wish to enter a children's hospital you will probably have to wait awhile. You will find all the information you want in the book mentioned.

(4) *Lecturer (E.B.)*—Apply to Miss Lamport, 52, St. John's Wood Road, N.W.

### Wants and Workers.

[The attention of correspondents is directed to the fact that "Helps in Sickness and to Health" (Scientific Press, 428, Strand) will enable them promptly to find the most suitable accommodation for difficult or special cases.]

Can anyone tell a poor nurse, who is partly paralysed, of a permanent home where she could be received for the payment of 10s. a week?

A permanent home also wanted for a man suffering from spinal disease who could pay from 7s. to 10s. a week. In both cases please reply to the Editor, 428, Strand.

Can you tell me where a girl of fifteen suffering from epileptic fits could be received free of charge, or for a small sum weekly? Reply to Sister Grace, care of the Editor, 428, Strand, W.C.

### A Book and its Story.

### "WITH AN AMBULANCE DURING THE FRANCO-GERMAN WAR, 1870-1871."*

War stripped of all glamour is presented in these pages, and the element of traditional romance which has centred round the battlefields of fiction is conspicuous by its absence. The book is simply the embodiment of a series of notes and jottings taken in spare moments during the Franco-German War, and from the standpoint of a close observer of the momentous scenes. The author, neither a warrior nor a writer, speaks to us with a sincerity of expression at once convincing.

"As I have tried to write down exactly what I witnessed," Dr. Ryan says, "these notes may help to afford some idea of what war really means—war, as a hard, practical fact—stripped of its poetry and pride of conquest, which are so attractive when seen in history."

A hatred of the misery war brings in its wake, and a keen sense of horrors during the period of havoc—such is the keynote of the volume; and all will readily admit that a greater acquaintance with these facts is made through the instrumentality of the writer's pen.

In 1870 Dr. Ryan was a young medical student in Dublin, and he, in company with most other Irishmen, felt enthusiastic interest at that epoch in the destinies of France. So ardent was his sympathy, that he desired to show it in some active manner. The idea of actually fighting in the French cause presented itself only as an unfeasible project, when the more feasible suggestion of joining an ambulance occurred to the youthful aspirant's mind, and he accordingly leaves his country to follow out his plan.

Several pages describe the difficulties which beset him in Paris of effecting this design; ultimately success crowned his endeavours, and a place was made for the young Ryan in the Anglo-American Ambulance, which had been organized by English and American surgeons in the French capital. The departure of the corps for the scene of action is thus given. "A death-like silence reigned throughout the crowd, as we were waiting for the final start. The Count de Flavigny came forward and addressed us in a long and eloquent speech, as he reminded us of the scenes upon which we were to enter; the cause we were to vindicate; the hardships we were likely to undergo; the good that each of us was bound in duty to perform; the sacrifice of every personal consideration, and even of our lives if necessary, in the grand and holy cause of the service of the wounded."

While they stood there, in the Champs Elysées, "a young girl, pretty, and elegantly dressed in deep morning," the author tells us, "stepped up and tried to address me; but she sobbed so much, that I could with difficulty understand what she said. After a long time she made her wish intelligible. Should her husband ever come across my path in a wounded condition, she charged me to be kind to him, and to bestow upon him particular care for her sake."

On Tuesday, August 30th, the ambulance reached Sedan, where orders were received for immediate departure to Cavignan, where hard fighting had been going on, and where, they were told, the field had been won by the French under Macmahon. At this point Napoleon is described as Dr. Ryan first saw him: "He had a tired, scared, and haggard appearance, and, besides looking thoroughly ill, seemed anxious and impatient." The great battle of Sedan was on the following day. Stationed on high ground above the scene of the attack, the ambulance had an opportunity in their leisure moments of witnessing the whole of the terrible drama enacted before them. The descriptions of the encounters of the two armies here, at Orleans, and elsewhere, are given in so rapid and vivid a manner, with such spontaneous a directness, that they stamp an historical value on these pages. From one scene of disaster to another we turn with amazing speed, to find the German army in the ascendancy.

What was the meaning of the repeated disastrous defeats of the French army? Dr. Ryan asks. And the answer he gives points out the particular bearing of the medical mind.

He traces the result of the encounters to moral and intellectual causes—causes which he asserts are still existent in France. The physique of the two opposing nations were in strong contrast the one to the other. Speaking of the stalwart appearance of the Prussian soldiers, he says: "A notable contrast, indeed, they presented to the stunted, nervous-looking, and worn-out French soldiers, who, however, it is only fair to add, were suffering from the effects of long exposure and privation, and whom we had seen at their worst. Still, there was a difference in the men themselves. What was the explanation of it? He that can reply to this question as the truth demands, and he alone, will explain why the French campaigns of 1870 and 1871 were such a dismal series of misfortunes. The breakdown of the commissariat, the peculation in high quarters, tho confused plans, and the military disorder must be ascribed to causes which were long in action before the French entered on their struggle with the Fatherland."

This stunted and undeveloped make of the youthful French levies, Dr. Ryan insists, were traceable to no other causes than the overtaxed vital energies on the part of the parents, and on the part of the men to the total neglect of their early moral training, and the absence of that education which tends to a manly development. These were the main causes of the crushing defeats following on each of the engagements described in the book before us.

The total unpreparedness of the French; the want of discipline among the soldiers, and command from the officers who were engaged in the war, is constantly pointed out. One French colonel confessed to Dr. Ryan that he had been six hours under fire without receiving any orders from his general, and many instances of want of military order intersperse the pages. All was confusion and disorder throughout the army.

On the other hand, the discipline on the German side was admirable. Their adversaries did not realize that they had to deal with an army the rank and file of which not only was composed of the muscle and sinews of the German people, but included their best brains also. Science and civilization have rarely been better expressed than in the great war of 1870. But there are instances given, also, of fine courage among the French, the story of the brave Chasseur d'Afrique being a notable example of the French soldier at his best.

If Dr. Ryan is critical of the shortcomings of the one army, so is he also of the other; the faults he lays to the charge of the Germans are by no means light ones. Of his own personal experience in the ambulance we have spoken insufficiently, because this should be read and followed in its every detail. But the horrors of war, the hunger, cold, and general privation, are brought home to us in this instance as no other writer has succeeded in doing. The whole book, as we have said, points to one great moral lesson on war.

Dr. Ryan contrasts, at some length, the antiseptic treatment of wounds of to-day in a highly favourable light compared with the treatment of 1870, and he insists that a great part of the exceptionally high rate of mortality could have been lessened, had the precautions of to-day been in existence twenty-five years ago.

* "With an Ambulance during the Franco-German War, 1870-1871." By Charles E. Ryan, F.R.C.S.I., M.R.C.P.I. (London: John Murray. 1896.)

### The Book World for Women and Nurses.

[We invite Correspondence, Criticism, Enquiries, and Notes on Books likely to interest Women and Nurses. Address, Editor, The Hospital (Nurses' Book World), 428, Strand, W.C.]

Infant Feeding by Artificial Means. By S. H. Sadler. Second Edition. (London: Scientific Press. 1896.)

This little work, which is now on the assured and dignified footing of a second edition, is presented in much the same form as when originally published; but the general appearance and get-up is much improved, and really excellent illustrations have been added. It contains, however, a special chapter on the history of infant feeding, a chapter which is well illustrated, and full of interesting facts regarding the practice of the ancients. "Infant Feeding" is for the most part a summary of the views of the chief authorities in Europe on this important branch of dietetics; for this reason it is a work well worthy of the attention of those engaged in the bringing up of children, as well as of those practitioners who have not the time or opportunity for collecting the information for themselves. If we may be allowed to make a few suggestions—in case, as is highly probable, a third edition may be called for—we would advise that an account of Dr. Rolch's scheme for preparing modified milk at milk laboratories should be embodied in the pages of "Infant Feeding," since the method has already proved useful in America, and is based on a sound and rational principle. The necessity for an antiscorbutic element in infant food, and the danger of employing for too long a period either condensed milks or other prepared food should also be insisted upon. The word "heat" as being the second essential for successful feeding should be changed for "temperature." When these few little points are borne in mind, the volume before us will be found as useful as it is attractive.

Benjamin Townson: A Record of a Doctor's Life and Work. By his Daughter. (London: S. W. Partridge and Co.)

The record of the life of many a general practitioner in the present day is one of unremitting labour and self-sacrifice. Benjamin Townson set an example of faithful work in his care of the large practice which fell to his lot in the busy town of Liverpool. A member of the Society of "Friends," and a deeply religious man, he practised what he preached, and busied himself in many charitable and philanthropic undertakings. An affectionate and kindly father, he was repaid by the whole-hearted love of his sons and daughters, and the home circle was a bright and happy one. Dr. Townson was a strong advocate of temperance, and in his own professional practice deprecated the use of alcohol, and laid much stress on the danger of rashly ordering stimulants. In 1883 Dr. Townson at length retired from active work to enjoy some well-earned rest, and after a most patiently-borne illness he died on August 21st, 1888, at the ripe age of seventy-seven, leaving behind him the memory of a worthy member of his noble profession, and a record of a stainless life of labour for others.

A Few Suggestions for the Management of Young Children. By Rev. R. H. and Mrs. Hart Davis and Hastings Gifford. To be obtained of Mr. W. Salmon, 54, London Street, Reading. Price 1d.

The management, both as regards the feeding and the moral training of young children, will always be a matter of great importance. The little pamphlet before us, which devotes itself to this subject, is a step in the right direction, clearly pointing out as it does the absolute necessity of commencing the training of the child from its very earliest infancy. The paragraphing is good, as thereby information can be easily and readily acquired, and the modest price of 1d. will place it within the reach of all.

### For Reading to the Sick.

GOOD FRIDAY.

**Motto.**

Love as strong as death.

**Verses.**

Follow to Calvary,
Tread where He trod,
He Who for ever was
Son of God. —*Edward Monro.*

Come, sinners, view the Lamb of God,
Wounded and dead, and bathed in blood!
Behold His side, and venture near,
The well of endless life is here. —*John Newton.*

See from His head, His hands, His feet,
Sorrow and love flow mingling down:
Did e'er such love and sorrow meet,
Or thorns compose so rich a crown?

Were the whole realm of Nature mine,
That were an offering far too small;
Love so amazing, so divine,
Demands my soul, my life, my all.

O teach us, Lord, to bear our Cross,
In meekness unrepining;
Where deepest go the nail-prints, there
The sweetest flowers are shining.
—*Henry N. Oxenham.*

Our pains are portioned to our powers—His hand may hurt, but cannot harm—
But if the Cross be on us laid, and our souls crowns of thorns be made,
Then, sure, 'twere best to bear the Cross, nor lightly fling the thorns behind,
Lest we grow happy—by the loss of what was noblest in the mind!
Here—in the ruin of my years—Master, I thank Thee through my tears—
Thou suffered'st here, and did'st not fail—Thy bleeding feet these paths have trod—
But Thou were strong, and I am frail; and I am man, and Thou art God.
How I have striven, Thou know'st! Forgive how I have failed, Who saw'st me strive. —*Lytton.*

**Reading.**

"And now we come to the great day to which all Lent has been pointing—Good Friday, at once the saddest and the most triumphant day that ever dawned upon the earth. Saddest, because of the shame and agony of His sufferings, and the inconceivable wickedness of mankind in inflicting them; triumphant, because in dying He conquered death, and overthrew him who had the power of death, even the devil.

"Picture the scene on Calvary. It is nearly three o'clock, the darkness is clearing away, light is returning, all is accomplished, Scripture fulfilled, everything finished, but Death delays!

"Death is the punishment of sin, but there is no sin in this Divine Sufferer, therefore Death dares not approach; he beholds nothing there that is his, Death cannot touch Him till Christ gives leave. Of all the evils which our Saviour took upon Him for our sakes, there now remains only the act of death for His voluntary endurance.

"It is three o'clock; the great Death has taken place, the great sacrifice is offered! The true peace offering, reconciling man to God in the sacramental stream which ever flows from His pierced side.

"Three o'clock! the Hebrews are offering the Pascal lamb in the Temple court; but it is to Calvary we must come, and with bended knees and adoring, thankful, humble hearts exclaim, 'Behold the Lamb of God, which taketh away the sin of the world.'"—*From "A Lent with Jesus."*

